# Dietary Habits and their Association with Metabolic Syndrome in a sample of Iranian adults: A population‐based study

**DOI:** 10.1002/fsn3.1918

**Published:** 2020-09-29

**Authors:** Elnaz Lorzadeh, Zohreh Sadat Sangsefidi, Masoud Mirzaei, Mahdieh Hosseinzadeh

**Affiliations:** ^1^ Nutrition and Food Security Research Center Shahid Sadoughi University of Medical Sciences Yazd Iran; ^2^ Department of Nutrition School of Public Health Shahid Sadoughi University of Medical Sciences Yazd Iran; ^3^ Yazd Cardiovascular Research Centre Shahid Sadoughi University of Medical Sciences Yazd Iran

**Keywords:** diet, dietary habits, metabolic syndrome

## Abstract

**Background:**

Central obesity, insulin resistance, dyslipidemia, and hypertension are the core components of metabolic syndrome (MetS) which is coincident with unhealthy dietary habits in the Middle‐Eastern countries. The aim of this study was to explore the association between dietary habits and MetS of the adult population living in Yazd Greater Area, Iran.

**Methods:**

This is a cross‐sectional study that uses the data of a population‐based cohort study on Iranian adults, known as Yazd Health Study (YaHS). The relationship between dietary habits and metabolic syndrome among adults (*n* = 2,896) was analyzed using multiple logistic regression method.

**Results:**

The prevalence of MetS among the participants was 32.2%. Outcomes from logistic regression examination show that breakfast consumption has a significant inverse effect on the occurrence of MetS after adjustment for age, education level, physical activity statue, history of chronic diseases, and smoking (odds ratio (OR) = 0.38, 95% confidence interval (CI) = 0.14, 0.97). This effect remains significant even after adjustment for body mass index (BMI) and reveals that odds of having MetS is 69% lower in breakfast consumers in contrast to nonconsumers (OR = 0.31, 95% CI = 0.11, 0.87). However, no significant relationship was observed between other dietary habits including consumption of sweetened drinks, sugar cubes, and fast foods and MetS after adjustment for all potential confounders.

**Conclusions:**

This study revealed that eating breakfast has an inverse relationship with metabolic syndrome. To find out stronger evidence in relation to dietary habits and MetS, more researches especially population‐based cohort studies are needed to be conducted.

## Graphical abstract

This study reveals that eating breakfast has an inverse relationship with metabolic syndrome. To find out stronger evidence in relation to dietary habits and MetS, more researches especially population‐based cohort studies are needed to be conducted.

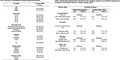

## 1 BACKGROUND

With an increased risk of diabetes mellitus (DM) and cardiovascular disease (CVD), which are mostly derived from metabolic syndrome (MetS) (Grundy et al., 2004), urgent strategies to prevent its global epidemic are demanded (Zimmet et al., 2001). Central obesity, insulin resistance, dyslipidemia, and hypertension are the core components of metabolic syndrome (Kirby et al., 2010). According to the international diabetes federation (IDF), the rapid increase of MetS has been estimated to be 20%–25% globally (O'neill & O'driscoll, 2015) which is parallel to the growing rate of DM, hypertension, cardiovascular disease and obesity (Zimmet et al., 2001). The prevalence of MetS in different studies varies, depending majorly on the used criteria of different definitions and their compositions (such as sex, age, race and ethnicity) (Cornier et al., 2008). For example, results of recent systematic review and meta‐analysis of studies revealed the overall estimation of MetS prevalence in Iran to be 36.9% based on the Adult Treatment Panel guidelines III (ATP III) criteria and 34.6% according to IDF (Amirkalali et al., 2015; Dalvand et al., 2017). However, regardless of the criteria used, it has been reported that the prevalence of metabolic syndrome around the world is rising drastically (Aguilar et al., 2015; Prasad et al., 2012).

Unhealthy diets and lifestyle are some of the risk factors contributed to MetS (Cox et al., 2013; Organization, 2013). Several studies have been conducted to explore the relationship between dietary habits and MetS (Azadbakht et al., 2005; Elwood et al., 2007; Esmaillzadeh et al., 2006; Lutsey et al., 2007; Panagiotakos et al., 2007; Ruidavets et al., 2007; Snijder et al., 2007; Wirfält et al., 2001). It has been reported that dietary habits that are more westernized can increase the chances of MetS (Wirfält et al., 2001), whereas diets rich in fruits and vegetables (Esmaillzadeh et al., 2006) might have a reduction effect.

Considering what has been mentioned above, emerging evidence reveals that higher consumption of sugar‐sweetened beverages consist of energy‐containing sweeteners such as sucrose, high fructose corn syrup or, fruit juice concentrates (Duffey & Popkin, 2008) is associated with increasing risks of MetS (Malik et al., 2010b) not only in the US diet (Popkin, 2010) but also in developing countries such as India and China (Bray, 2007). These findings also have been confirmed by a meta‐analyses of cohort studies (Malik, et al., 2010a).

Moreover, studies suggest that consumption of out‐of‐home meals and energy‐dense fast foods (Bahadoran et al., 2013) and snacks, which contain highly processed meat, total fat (saturated and trans‐fatty acids), and refined carbohydrates (Esmaillzadeh & Azadbakht, 2011) as well as sodium, can cause postprandial metabolic disorders such as dyslipidemia, subclinical inflammatory, and oxidative stress and thereby increase the occurrence of MetS (Devaraj et al., 2008; Marangoni et al., 2019; Mendoza et al., 2007), all of which leads to elevated fasting insulin levels and metabolic syndrome in adults (Mendoza et al., 2007). In regard to breakfast habits and MetS, there are some controversial results. For example, those who habitually do not consume breakfast are at higher risk for skipping other meals, snacking, having a sedentary lifestyle and being obese or overweight (Rampersaud et al., 2005; Utter et al., 2007). Eating breakfast is associated with higher overall diet quality (Carson et al., 1999), and since it is satiating, it can reduce the total energy intake of the day and cause a beneficial impact on improving metabolic parameters (de Castro, 2004; Ruxton & Kirk, 1997). On the contrary, a study suggested that skipping breakfast alone did not have any significant association with MetS prevalence (Kutsuma et al., 2014).

Despite previous studies that have investigated metabolic syndrome's association with dietary habits, there are only a limited number of them conducted in the Middle‐eastern countries where nutritional transition to westernized eating habits has caused an increase in the prevalence of overweight and obesity. This study aims to explore the association between dietary habits and MetS of a large population of adults living in Yazd Greater Area in Iran.

## 2 MATERIALS AND METHODS

### 2.1 Study population and data collection

Yazd Health Study (YaHS) data were collected in 2014–2015. Its recruitment phase data were used for this cross‐sectional study. YaHS was initiated among the adult population living in Yazd Greater Area, with 10,000 participants between the ages of 20–70 years old. Established in November 2014 and containing two phases, YaHS at first randomly selected 200 clusters according to the city postcodes. Then, the interviewers set up a meeting time with the residences at each of their assigned addresses. At last, as the interviewers visited the neighborhood of the first addressee based on the study protocol to fill in the questionnaire forms (Masoud Mirzaei et al., 2018). A validated questionnaire consists of 300 questions including the following sections were filled by trained interviewers; (a) demographics, (b) physical activity, (c) sleep quality and quantity, (d) mental health, (e) past medical history of chronic disease and surgical operations, (f) dental health, (g) history of accidents, (h) dietary habits, (i) occupation and social life, (j) traditional medicine, (k) smoking habit and drug addiction, (l) women's health, and (m) quality of life (Masoud Mirzaei et al., 2018). Overall, almost 40% of the YaHS participants gave consent for blood sampling and biochemical assessment in the first phase. Thus, only approximately 3,748 persons had data on biochemical assessment and metabolic Syndrome. However, the difference in age group and sex of those who gave consent for blood sampling was not different from the rest. Out of 3,748 available cases, the subjects with the following conditions were excluded, having a history of cardiovascular disease, diabetes, and cancers. Eventually, 2,896 participants entered the current study.

Demographics data, anthropometrics (weight, height, and waist circumference), socioeconomic status, education, physical activity, tobacco smoking, and dietary habits in addition to biochemical assessments including; fasting blood glucose, triglyceride, and HDL cholesterol levels were also carried out.

The present study was approved by the Ethics Committee of Shahid Sadoughi University of Medical Science (IR.SSU.MEDICINE.REC.1395.287). Written informed consent was obtained from all the participants.

In general, all the required information on participants’ dietary habits, anthropometric indices measurements, blood test results, blood pressure measurement and confounding factors consisting of socioeconomic status, history of chronic diseases and physical activity status was extracted and merged from YaHS database (Mirzaei et al., 2018).

### 2.2 Dietary assessment

We aimed at obtaining accurate information on some dietary habits noted by participants via a questionnaire (Mirzaei et al., 2018). These items were as follows: (a) sweetened drinks (fruit juices, artificially or homemade sweetened beverages) with frequency consumption of not at all, less than once per week, once or more per week, (b) fast foods consumption with frequency of not at all or few times per year, 1–3 times per months, once or more per week, (c) breakfast consumption as not at all in frequency, once per week or more than once per week, (d) sugar cubes with the serving intake of not at all, 1–2 cubes per day and more than 2 cubes per day.

### 2.3 Diagnosis of metabolic syndrome

National Cholesterol Education Program and Adult Treatment Panel III criteria (NCEP) present the definition of MetS in the current study (Chackrewarthy et al. 2013; Grundy et al., 2005; Huang, 2009). To be diagnosed with at least 3 risk factors out of the five following cases, means a participant has metabolic syndrome if: (a) triglyceride (TG) above 150 mg/dl or consuming triglyceride‐lowering agents (hyperglycemia); (b) high‐density lipoprotein‐cholesterol (HDL‐C) level of less than 40 mg/dl in men and less than 50 mg/dl in women or any kind of pharmacological treatment; (c) systolic blood pressure above 130 mmHg and diastolic blood pressure above 85 mmHg (hypertension); (d) fasting blood glucose above 100 mg/dl or usage of pharmacological treatment as control from blood sugar; and (e) and waist circumference (WC) above 91.5 cm in men and 85.5 cm in women (adopted for only Iranian population) (Esteghamati et al., 2008).

### 2.4 Evaluation of anthropometric indices

Weight was recorded with participants wearing lightweight clothing and no shoes using a digital scale (Model BF511, Omron Co. Karada Body Scan, Osaka, Japan) with a precision of 0.1 kg. A nonstretchable tape meter with the precision of 0.5 cm was used to measure height with lightweight clothing and no shoes and with their heels, hip, shoulder, and head touching the wall. Body mass index (BMI; weight [kg]/height [m2]) was calculated from measured height and weight. Waist circumference was also measured by tape in the horizontal plane midway between the iliac crest and the rib cage with a precision of 0.1 cm.

### 2.5 Blood pressure measurement

Systolic and diastolic blood pressures were measured in the sitting position by an automatic digital blood pressure monitor (Riester Germany). Each measurement was repeated for two times after every 5‐min interval.

### 2.6 Laboratory data

Data on serum level of fasting blood glucose (FBG) (mg/dl), total cholesterol, triglycerides, low‐density lipoprotein‐cholesterol (LDL‐C), and high‐density lipoprotein‐cholesterol (HDL‐C) were measured and collected. The equipment used in this study included a calibrated Ciba Corning auto‐analyzer device (Ciba Corp., Basle, Switzerland (and Pars Azmoon company kits to assess fasting blood glucose and triglyceride after centrifuging and bionic kits as an analyzer for HDL cholesterol.

### 2.7 Physical activity assessment

For physical activity ascertainment, the Iranian Short Version of International Physical Activity Questionnaire (IPAQ) was given to the participants (Hu, 2002; Moghaddam et al., 2012). Physical activity levels of each participant were categorized as active and inactive based on the guideline of the short form of IPAQ (Mintie et al., 2020).

### 2.8 Statistical analysis

All statistical analyses were performed using SPSS version 24 (SPSS Inc.), and to show the qualitative variables, they went under a frequency and percentage discretionary. Multiple logistic regression was used to examine the association between dietary habits and MetS. The lowest frequency or serving indicates a reference for all the models and adjustments of the confounding factors which are as follows: Age (20–29, 30–39, 40–49, 50–59, 60–69 years), educational level (secondary school and lower, high school, diploma and graduate diploma, bachelors, masters, and Ph.D.), history of chronic diseases (yes/no, including: hypertension, diabetes, cardiovascular disease, cancer, depression, dyslipidemia), smoking history (yes/no), physical activity level (inactive and active), and BMI (continuous).

## 3 RESULTS

Table 1 shows the general characteristics of the study population. A majority of participants (53.2%) as illustrated were females. Furthermore, a high percentage of the participants (24.7%) were between the ages of 40–49 years old and had secondary school or lower education (53.5%). The smoking status also demonstrates that a high percentage of the volunteers did not smoke (90.0%) and were married (86.9%). Moreover, 51.8% of the participants were active in their physical activity statue.

**Table 1:** Distribution of all participants according to general characteristics

Variables	Total (*n* = 2,896) *N* (%)
Sex	
Male	1,352 (46.8%)
Female	1,534 (53.2%)
Age (years)	
20–29	541 (18.8%)
30–39	639 (22.2%)
40–49	713 (24.7%)
50–59	560 (19.4%)
60–69	429 (14.9%)
Education level	
Secondary school and lower	1,534 (53.5%)
Diploma and Graduate diploma	877 (30.6%)
Bachelors	389 (13.6%)
Masters and PhD	66 (2.3%)
Smoking	
Yes	185 (6.6%)
Sometimes	55 (2.0%)
Quitted	43 (1.5%)
No	2,533 (90.0%)
Marital status	
Married	2,505 (86.9%)
Single	274 (9.5%)
Widowed	89 (3.1%)
Divorced	15 (0.5%)
Physical activity	
Inactive	1,396 (48.2%)
Active	1,500 (51.8%)

The prevalence of the metabolic syndrome among the participants was 32.2% as demonstrated in Table 2. Additionally, the plurality of the participants (38.6%) was overweight (BMI = 25–29.9). Moreover, 38.8% met the criterion for hypertriglyceridemia (plasma TG higher than 150 mg/dl) and 29.3% had FBG higher than 100 mg/dl, while 65.8% had low levels of HDL‐C (˂40 mg/dl in men and ˂50 mg/dl in women). Furthermore, abdominal obesity which is considered as waist circumference >91.5 cm for men and 85.5 cm for women afflicted 65.1% of the study population. It was also found that 45.5% of the subjects suffered from high blood pressure (130/85).

**Table 2:** Distribution of participants according to metabolic syndrome (MetS) and its components

Variables	Total (*n* = 2,896) *N* (%)
BMI (kg/m^2^)	
Low weight (<18.5)	80 (2.9%)
Normal (18.5–24.9)	863 (31.3%)
Overweight (25–29.9)	1,062 (38.6%)
Obesity (30–39.9)	671 (24.4%)
Extreme Obesity (≥40)	77 (2.8%)
Triglyceride status (mg/dl)	
Lower than 150	1768 (61.2%)
Equal to or higher than 150	1,119 (38.8%)
Fasting Plasma glucose status (mg/dl)	
Lower than 100	2,044 (70.7%)
Equal to or higher than 100	849 (29.3%)
Waist Circumference status (cm)	
91.5> for men and 85.5> for women	1,001 (34.9.%)
91.5≤ for men and 85.5≤ for women	1,870 (65.1%)
HDL status (mg/dl)	
40< for men and 50< for women	1,900 (65.8%)
40> for men and 50 >for women	986 (34.2%)
Hypertension status (mmHg)	
No	1,571 (54.5%)
Yes	1,312 (45.5%)
MetS	
No	1,940 (67.8%)
Yes	920 (32.2%)

Furthermore, in Table 3 after adjusting the confounders, results of the logistic regression examining the association among those who consume breakfast for once per week compared to those who completely skip breakfast shows a significant relationship with odds of metabolic syndrome, which means that these subjects have 62% lesser chance of MetS (odds ratio (OR) = 0.38, 95% confidence interval (CI) = 0.14–0.97). This effect remains significant even after adjustment for BMI and reveals that odds of MetS is 69% lower in breakfast consumption as once per week in contrast to nonconsumption (OR = 0.31, 95% CI = 0.11–0.87).

**Table 3:** Multivariable‐adjusted odds ratios (95% CI) for metabolic syndrome across different frequencies or servings for various dietary habits in a sample of Iranian adults

Dietary habits^a^	Metabolic syndrome				
	Multivariable‐adjusted^b^		Multivariable + BMI^c^		
	OR	95% CI	OR		95% CI
Sweetened drinks					
Not at all	Reference		Reference		
Lower than once per week	0.93	0.71–1.22	0.97		0.74–1.28
Once or more per week	0.98	0.76–1.27	1.07	0.82–1.40	
Fast foods					
Not at all or few times per year	Reference		Reference		
1–3 times per month	1.02	0.81–1.28	0.93		0.73–1.18
Once or more per week	0.92	0.62–1.36	0.88		0.58–1.31
Breakfast					
Not at all	Reference		Reference		
Once per week	0.38*	0.14–0.97*	0.31*		0.11–0.87*
More than once per week	0.74	0.40–1.37	0.36		0.36–1.32
Sugar cubes					
Not at all	Reference		Reference		
1–2 cubes per day	1.01	0.71–1.45	1.03		0.71–1.49
More than 2 cubes per day	1.05	0.76–1.45	1.09		0.78–1.52

^a^ Dietary habits for all items were presented as frequency of consumption except for sugar cubes which presented as serving of intake.

^b^ Adjusted for age (20–29, 30–39, 40–49, 50–59, 60–69 years), education level (Secondary school and lower, High school, Diploma and Graduate diploma, Bachelors, Masters and PhD), physical activity level (active and inactive), history of chronic diseases (hypertension, diabetes, cardiovascular disease, cancer, depression and dyslipidemia), smoking (yes/no).

^c^ Adjusted for age (20–29, 30–39, 40–49, 50–59, 60–69 years), education level (Secondary school and lower, High school, Diploma and Graduate diploma, Bachelors, Masters and PhD), physical activity level (active and inactive), history of chronic diseases (hypertension, diabetes, cardiovascular disease, cancer, depression, and dyslipidemia), smoking (yes/no) and BMI.

* Significance level was considered as *p* < .05.

Nevertheless, no significant association has been witnessed between other dietary habits including consumption of sweetened drinks, sugar cubes, and fast foods with MetS after adjusting for potential confounders.

## 4 DISCUSSION

In the current study, consumption of breakfast can significantly contribute to a low chance of MetS. Conversely, consumption of sweetened beverages, fast foods, and sugar cubes hase been shown to have a nonsignificant impact on the occurrence of MetS. These relations between dietary habits and MetS were maintained after adjusting for potential confounders such as age, education level, and physical activity level, the history of chronic diseases, smoking, and BMI.

Many studies suggest that eating breakfast can associate with overall better diet quality and a healthy lifestyle (de Castro, 2004; Ruxton & Kirk, 1997; Song et al., 2005). It has been also reported that individuals who eat breakfast frequently have a lower risk of an array of metabolic outcomes comparing to their peers who infrequently or never eat breakfast (Odegaard et al., 2013). Many studies suggest that breakfast intake is beneficial to metabolic health which is in the same line as our findings (Cayres et al., 2016; Odegaard et al., 2013). This, as provided by a spectrum of research evidence could be for the reason that eating breakfast plays an important role in factors related to appetite and glucose, insulin and lipid metabolism (Klok et al., 2007; Odegaard et al., 2013; Schlundt et al., 1992). It has been discussed in some studies that poor breakfast habits can be a part of an unhealthy lifestyle which predicts metabolic syndrome (Wennberg et al., 2015). Several pieces of evidence suggest that breakfast intake beneficial effects depend on the type of breakfast (Cho et al., 2003; Deshmukh‐Taskar et al., 2010; O'Neil et al., 2014). It has also been demonstrated that a healthy breakfast (such as a fruit smoothie or milk breakfast) may influence subsequent dietary intake (McCartney et al., 2019). Iranian breakfast usually consists of bread, cheese or, butter or egg and drinks such as tea and milk (Ahadi et al., 2016) which can have a positive effect on metabolic factors. As has been reported in recent studies whole grains (Tieri et al., 2020) and low‐fat dairy (Godos et al., 2020) consumption as common components of healthy breakfast can be beneficial.

On the contrary, controversial outcomes report that a recommendation to eat or skip breakfast may not affect body weight (Dhurandhar et al., 2014). It has been reported that breakfast intake can potentially improve energy balance along with insulin and glucose levels thus leading to satiety and lesser weight (Freitas Júnior et al., 2012). Breakfast consumers are also more likely to have better overall dietary quality along with following dietary recommendations more than nonconsumers (Rampersaud et al., 2005). Obesity and high fasting glucose, the components of the metabolic syndrome and higher levels of total cholesterol (TC) and LDL‐C are also evident after skipping breakfast completely compared to regular breakfast eaters (Farshchi et al., 2005). According to a population‐based cohort study, MetS prevalence grows with the continuation of omitting breakfast or poor breakfast habits (Wennberg et al., 2015).

Furthermore, we found no association between the consumptions of sweetened beverages and sugar cubes and MetS in our population. Inconsistent to our outcomes, a few studies suggest no significant association between sweetened beverages consumption and a higher risk of metabolic syndrome and its components (Johnson et al., 2007; Khosravi‐Boroujeni et al., 2012; Valente et al., 2011). A prospective study also showed that sweetened drinks consumption was not associated with the incident of metabolic syndrome in a middle‐aged population (Lutsey et al., 2008). This, however, is in contrast with several other studies that report sugar‐sweetened drink intake has a significant effect on metabolic syndrome (Malik et al., 2006; Rivera et al., 2008; Woodward‐Lopez et al., 2011). Sweetened beverages that were included in this study consist of artificially or homemade beverages. Sweetened beverages in Iran are usually of plant origin which contains beneficial compounds such as polyphenols which are inversely associated with MetS and its components (Sohrab et al., 2013). This issue as well as different in confounders adjusted in the analysis could be a source of contradiction in this study and others because some studies did not adjust for BMI which is an important variable in relation between dietary habits and MetS.

Additionally, we could not find any significant association between fast‐food intake and metabolic syndrome. In contrast with our findings, some studies suggest that regular fast‐food consumption has an irreparable effect on general health and can increase the risk of obesity, insulin resistance and other metabolic abnormalities (Duffey et al., 2009; Pereira et al., 2005; Rudolph et al., 2007). However, there is not a definitive agreement on ‘fast foods’ definition (Glanz et al., 1998) and differences in ingredients of the fast foods in the different studies could have caused discrepancies in the outcomes. Moreover, fast‐food consumption in our study was generally low (most of the study population (60.6%) did not consume fast foods at all or the intakes were only a few times per year).

This study contains a few limitations. First of all, the design of this study is cross‐sectional which does not provide a liable source of causality. Second, the data on dietary assessments were based on self‐reported questionnaires. And third, the impact of confounders could not be fully controlled due to unknown or unmeasured factors. Strength points suggest that this study investigates the impact of dietary habits on metabolic syndrome among a relatively large sample in a Middle‐Eastern country. A large extent of confounders that might affect the metabolic syndrome or its components has been also considered and controlled.

## 5 CONCLUSIONS

In conclusion, this study provides evidence that there is in fact an inverse relationship between breakfast intake and metabolic syndrome. No significant association has been seen between other dietary habits and MetS. However, to find out stronger evidence in relation between dietary habits and metabolic syndrome, more studies especially population‐based cohort researches need to be conducted.

## CONFLICT OF INTEREST

The authors declare that they have no conflict of interest.

## ETHICAL APPROVAL

The present research was approved by the Ethics Committee of Shahid Sadoughi University of Medical Science (IR.SSU.MEDICINE.REC.1395.287).

## INFORMED CONSENT

Written informed consent was obtained from all study participants.

## CONSENT FOR PUBLICATION

Not applicable.

## Data Availability

All data generated or analyzed during this study has been included in this published article.
